# Toxicity and cytopathology mediated by *Bacillus thuringiensis* in the midgut of *Anticarsia gemmatalis* (Lepidoptera: Noctuidae)

**DOI:** 10.1038/s41598-019-43074-0

**Published:** 2019-04-30

**Authors:** Bárbara Monteiro de Castro e Castro, Luis Carlos Martinez, Sergio Guedes Barbosa, José Eduardo Serrão, Carlos Frederico Wilcken, Marcus Alvarenga Soares, Antonio Alberto da Silva, Amélia Guimarães de Carvalho, José Cola Zanuncio

**Affiliations:** 1 Departamento de Fitotecnia, Universidade Federal de Viçosa, Viçosa, Minas Gerais, 36570-900 Brazil; 2 Departamento de Biologia Geral, Universidade Federal de Viçosa, Viçosa, Minas Gerais 36570-900 Brazil; 3 Departamento de Engenharia Florestal, Universidade Federal de Viçosa, Viçosa, Minas Gerais 36570-900 Brazil; 4 Departamento de Proteção Vegetal, FCA/UNESP (São Paulo State University) – Campus de Botucatu, Botucatu, São Paulo 18610-307 Brazil; 5 Departamento de Agronomia, Universidade Federal dos Vales do Jequitinhonha e Mucuri, Diamantina, Minas Gerais 39100-000 Brazil; 6 Departamento de Entomologia/BIOAGRO, Universidade Federal de Viçosa, Viçosa, Minas Gerais 36570-900 Brazil

**Keywords:** Mechanisms of disease, Entomology

## Abstract

Bioinsecticides and transgenic plants, based on *Bacillus thuringiensis* (Bt) toxins are important when managing *Anticarsia gemmatalis* Hübner (Lepidoptera: Noctuidae), a soybean defoliator pest. The interaction of these toxins with the caterpillar’s midgut cells determines their efficacy as an insecticide. The objective was to evaluate the toxicity of *B. thuringiensis*, subsp*. kurstaki* strain HD-1 and cytopathological changes mediated by these bacterial toxins in the midgut of *A. gemmatalis* caterpillars. Insecticidal efficacy was determined by calculating lethal concentration values (LC_25_, LC_50_, LC_75_, LC_90_ and LC_99_) in the laboratory. Midgut fragments from *A. gemmatalis* were extracted after bacterial ingestion and evaluated by light, transmission electron and confocal microscopy. The Bt median lethal concentrations showed toxicity [LC_50_ = 0.46 (0.43–0.49) mg mL^−1^] to fourth instar *A. gemmatalis* caterpillars after 108 hours. Bt induces severe cytotoxicity to *A. gemmatalis* midgut epithelial cells with increasing exposure over time, causing cellular disorganization, microvillus degeneration, cell fragmentation and protrusion, peritrophic membrane rupture, and cell vacuolization. The cell nuclei presented condensed chromatin and an increase in lysosome numbers. Apoptosis occurred in the midgut cells of caterpillars exposed to Bt. A regenerative response in *A. gemmatalis* caterpillars was observed 8 hours after exposure to Bt, however this response was not continuous. Toxins produced by Bt are harmful to *A. gemmatalis* at median concentration with structural damage and death of the midgut epithelial cells of this insect.

## Introduction

The velvetbean caterpillar, *Anticarsia gemmatalis* Hübner (Lepidoptera: Noctuidae) is the main defoliator on soybean plants (*Glycine max* L. Merrill, Fabaceae)^1^. In Brazil, this pest occurs throughout the year, especially in the vegetative phase of plants and its control is realized mainly with synthetic insecticides^2–4^. Integrated Pest Management (IPM) programs aim at reducing the use of chemicals in pest control^1^ due to the negative effects of these products on non-target organisms^5,6^ and on the environment^7,8^. Biological insecticides, such as *Bacillus thuringiensis* (Bt) Berliner (Bacillaceae) strains, specific to target pests, with no toxic effects on other animals or the environment^9^ are an alternative to chemical control^10,11^. The wide Bt strain and toxin variety allow the production of bioinsecticides and the development of transgenic plants^12^.

*Bacillus thuringiensis* is a gram-positive, rod-shaped bacterium that produces insect-toxic proteins during sporulations^13,14^. After ingestion, the toxin crystals are solubilized due to the alkaline pH in the caterpillar midgut and their protoxins were activated by intestinal proteases. These protoxins bind to specific receptors in the microvilli of the midgut columnar cells, forming pores in the plasma membrane, causing cell lysis and insect death^15–18^.

The interaction of Bt toxins with the midgut of caterpillars determines its efficacy as an insecticide^19^, since the insect digestive tract is a physical and chemical barrier against invasive pathogens. The *A. gemmatalis* midgut is the largest portion of its digestive tract, having an epithelium consisting of four cell types: columnar or digestive cells responsible for the secretion of digestive enzymes and absorption^20^; goblet cells responsible for ionic homeostasis and absorption^20,21^; regenerative cells responsible for cell turnover^22–24^ and endocrine cells positioned as isolated cells at the baseline of the epithelium^25^ responsible for endocrine function^20^.

Columnar and goblet cell alterations and regenerative cell reductions are reported in the midgut of Lepidoptera *Plodia interpunctella* Hübner (Pyralidae)^26^, *Epiphyas postvittana* Walker (Tortricidae)^27^, *Bombyx mori* L. (Bombycidae)^28^ and *Alabama argillacea* Hübner (Noctuidae)^19^ when they were exposed to Bt. It is possible that every insecticidal protein affects midgut epithelial cells in a unique way, as there are many potential routes to cause midgut epithelial cell death. Bt, subsp. *kurstaki* strain HD-1, parasporal bodies are most used to control caterpillars^29,30^. In this study, we determined toxicity and cytopathological changes mediated by these bacterium toxins in the midgut of *A. gemmatalis* caterpillars.

## Results

### Toxicity

**Figure 1:**
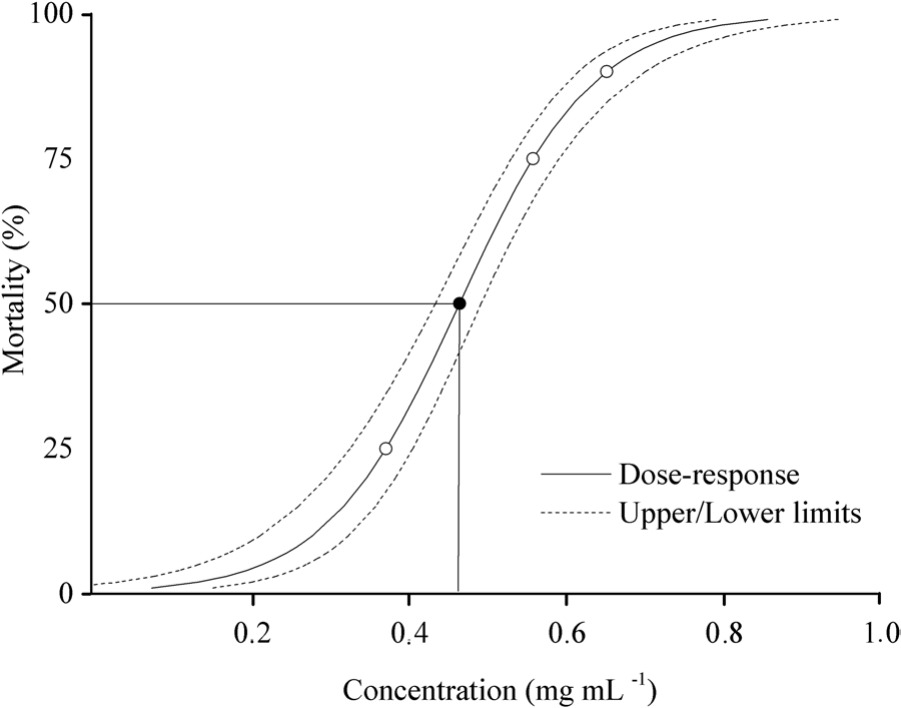
Mortality and upper and lower limits for fourth instar *Anticarsia gemmatalis* (Lepidoptera: Noctuidae) caterpillars exposed to different concentrations of *Bacillus thuringiensis* subsp*. kurstaki* strain HD-1.

The Bt lethal concentrations (X^2^ = 90.27, df = 5, *P* < 0.001) (Table 1) showed toxicity [LC_50_ = 0.46 (0.43–0.49) mg mL^−1^] to fourth instar *A. gemmatalis* caterpillars (Fig. 1). The mortality of *A. gemmatalis* caterpillars, by Bt toxins, depends on the bioinsecticide concentration and the exposure time, being 100% for those exposed to the highest concentration of Bt (3.2 mg mL ^−1^) and less than 1% in the control after 108 h of exposure.

**Table 1:** Lethal concentrations of *Bacillus thuringiensis* subsp.

^1^LC	^2^EV	^3^CI	^4^ *X* ^2^
25	0.37	0.32–0.40	90.27
50	0.46	0.43–0.49	
75	0.56	0.53–0.59	
90	0.65	0.61–0.70	
99	0.86	0.79–0.94	

Concentrations of ^1^LC_25_, LC_50_, LC_75_, LC_90_ and LC_90_ cause 25, 50, 75, 90 and 99% mortality; ^2^EV estimated value (mg mL^−1^), ^3^CI confidence interval (mg mL^−1^) ^4^X^2^, chi-square value for lethal concentrations and fiducial limits based on a logarithmic scale of significance level *P* < 0. 0001.

*kurstaki* strain HD-1 to fourth instar *Anticarsia gemmatalis* (Lepidoptera: Noctuidae) caterpillars.

### Histopathology

**Figure 2:**
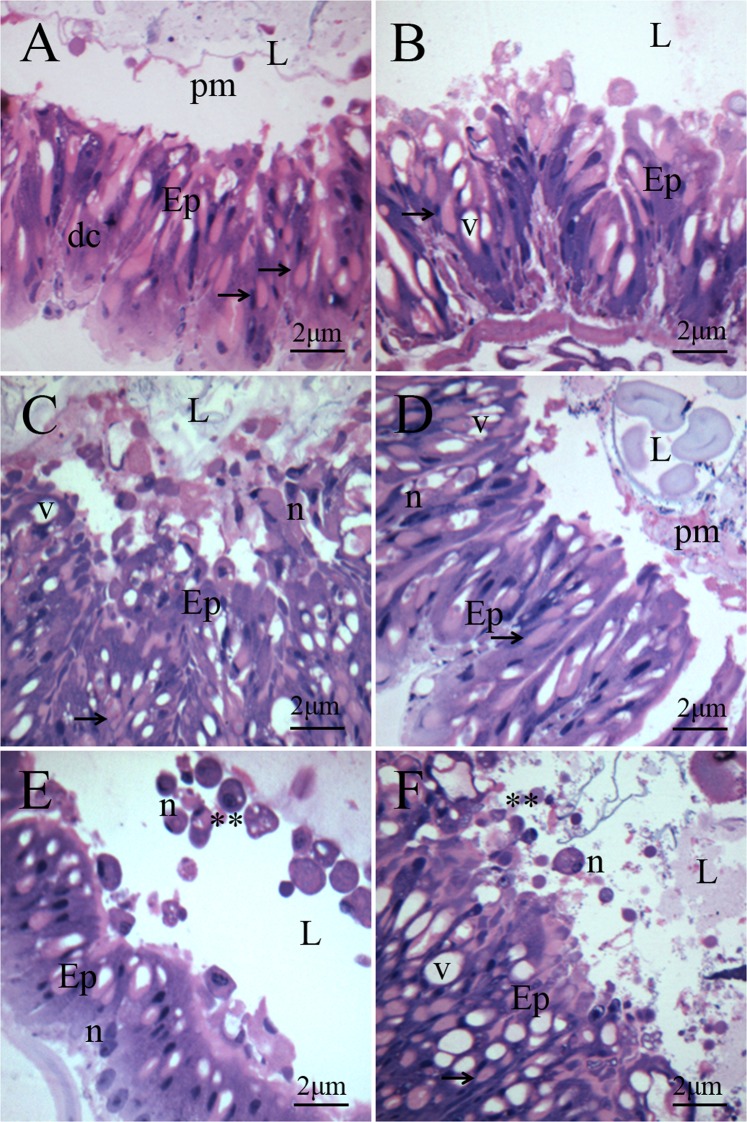
Midgut histological sections of fourth instar *Anticarsia gemmatalis* (Lepidoptera: Noctuidae) caterpillars not exposed to *Bacillus thuringiensis* subsp*. kurstaki* strain HD-1 (**A**) showing epithelium (Ep) with digestive cells (dc), goblet cells (setae) and preserved peritrophic membrane (pm) in the lumen (L) and of caterpillars exposed to Bt after 2 h (**B**), 4 h (**C**), 8 h (**D**), 16 h (**E**) and 32 h (**F**) the ingestion showing digestive cells (dc) with vacuoles (v), nuclei with condensed chromatin (n) and fragments of cells (**) released in lumen (L).

The *A. gemmatalis* caterpillar midgut not exposed to Bt presented epithelium composed of high columnar cells, goblet cells and evident peritrophic matrix. The cytoplasm of columnar and goblet cells had few vacuoles, vesicles and small granules. The nucleus was elongated, occupying the medial-basal cell portion, predominantly with decondensed chromatin (Fig. 2A).

**Figure 3:**
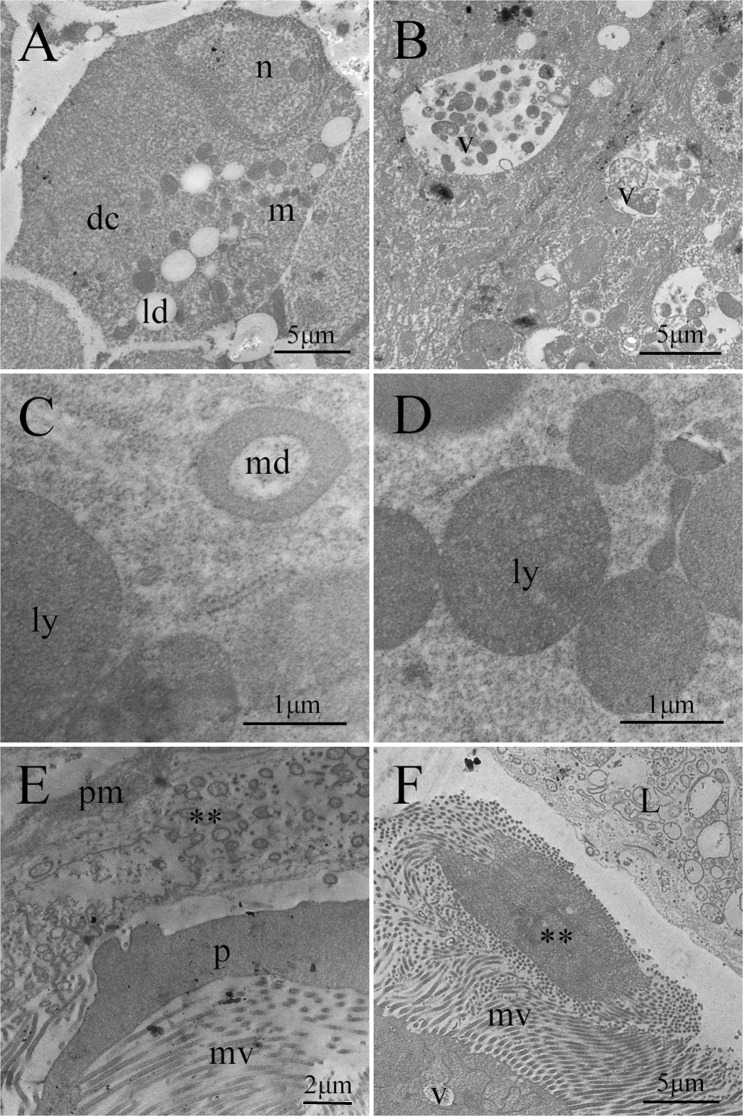
Midgut transmission electron microscopy of fourth instar *Anticarsia gemmatalis* (Lepidoptera: Noctuidae) caterpillars not exposed to *Bacillus thuringiensis* subsp*. kurstaki* strain HD-1 (**A**) showing digestive cells with a cytoplasm rich in mitochondria (m) and lipid droplets (ld) and of Bt exposed caterpillars (**B**–**F**) for 32 h showing digestive cells (dc) with vacuoles with cellular debris (v), lysosomes (ly), donut-shaped mitochondria (md), peritrophic membrane (pm) microvilli (mv), apical cell protrusions (p) some liberated (**) in the lumen (L).

Histological changes were observed in the midgut of *A. gemmatalis* caterpillars two hours after exposure to Bt (Figs 2B–3F). The epithelium presented irregular shapes, cellular degeneration and cellular fragments started in the lumen. The vacuolization of the cytoplasm was high and the peritrophic membrane was ruptured (Fig. 2B). At 4 h of exposure to the entomopathogen, the amount and size of the vacuoles increased, occupying much of the cell (Fig. 2C). A progressive increase of nuclei with condensed chromatin and cell fragments being released in the midgut lumen were observed within the 4–32 h interval (Fig. 2C–F).

### Ultrastructure

The midgut cell ultrastructure of *A. gemmatalis* caterpillars fed on non-Bt diet was well organized with dense cytoplasm and intact plasma membrane (Fig. 3A).

Midgut cells of caterpillars fed on Bt contaminated diet presented changes. Increased cytoplasm vacuolization and large autophagic vacuoles was observed (Fig. 3B). Donut-shaped mitochondria and numerous lysosomes were found in the intestine of toxin exposed insects (Fig. 3C,D). The microvilli were degenerated (Fig. 3E). Cellular protrusions and cell content fragmentation were observed in the midgut lumen (Fig. 3E,F).

### Immunofluorescence

**Figure 4:**
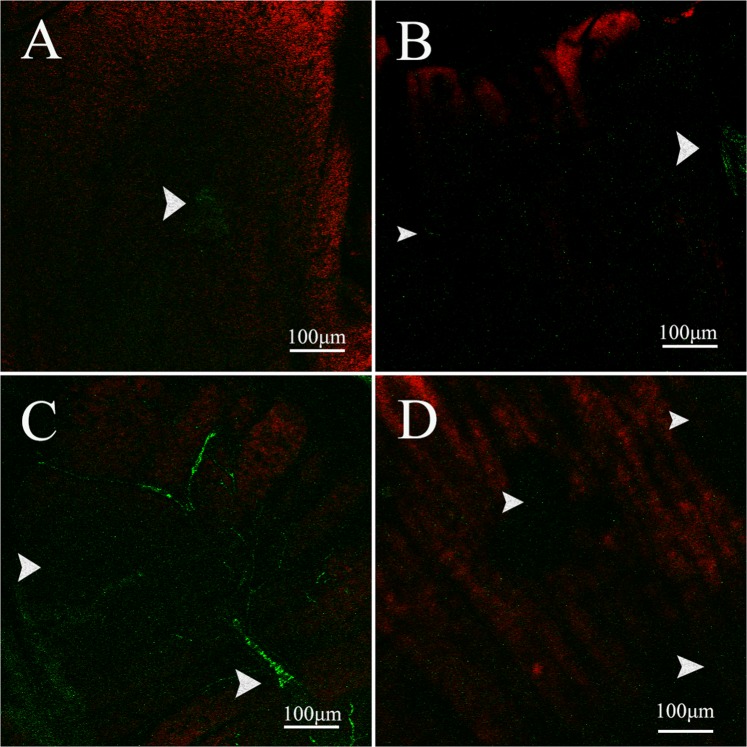
Immunofluorescence of *Anticarsia gemmatalis* (Lepidoptera: Noctuidae) midgut using the caspase-3 antibody (green - arrows). Sections of the caterpillars intestine not exposed to bacteria (**A**) and fed on *Bacillus thuringiensis* subsp*. kurstaki* strain HD-1 contaminated diet after 8 h (**B**), 16 h (**C**) and 32 h (**D**).

Cleaved caspase-3 was randomly distributed in the *A. gemmatalis* caterpillar midgut exposed or not to Bt (Fig. 4), but an increase of this protease was observed in the midgut of caterpillars at 8, 16, and 32 h after exposure to Bt (Fig. 4B–D).

**Figure 5:**
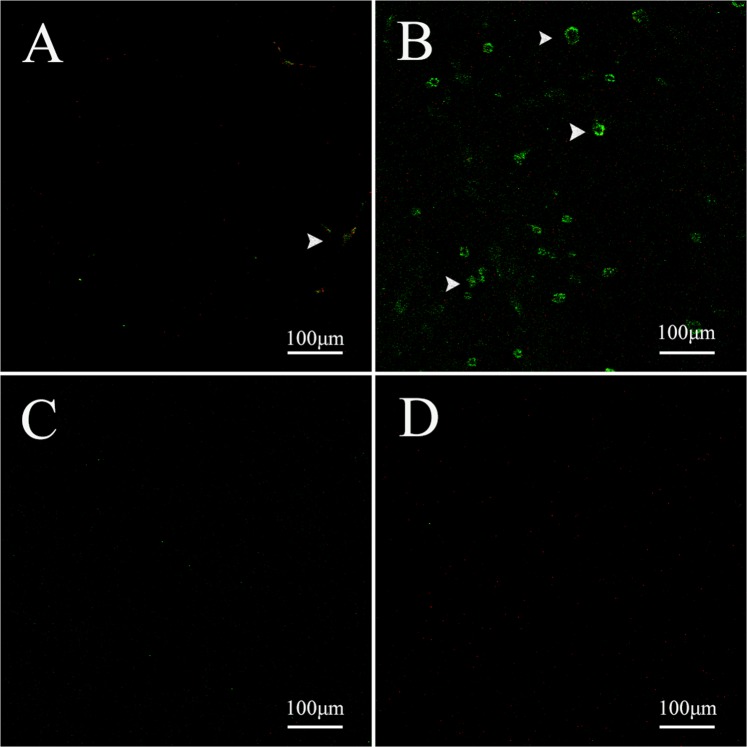
Immunofluorescence of *Anticarsia gemmatalis* (Lepidoptera: Noctuidae) midgut using the fosfo-histona H3 (PH3) antibody (green - arrows). Sections of the caterpillars intestine not exposed to bacteria (**A**) and fed on *Bacillus thuringiensis* subsp*. kurstaki* strain HD-1 contaminated diet after 8 h (**B**), 16 h (**C**) and 32 h (**D**).

An increase in the number of proliferating cells in the midgut of *A. gemmatalis* caterpillars was observed at 8 h after Bt exposure. However, this regenerative response was not observed at 16 and 32 h following bioinsecticide ingestion (Fig. 5).

## Discussion

*Anticarsia gemmatalis* susceptibility to Bt confirms bacterium efficacy when controlling this pest, however, this can vary according to the insect species^31,32^. *Spodoptera frugiperda* J. E. Smith^33^, *Helicoverpa armigera* Hübner^34^ and *Spodoptera litura* Fabricius^35^ (Lepidoptera: Noctuidae) are susceptible to different Bt concentrations. The mortality of *A. gemmatalis* caterpillars, due to Bt toxins, depends on the bioinsecticide concentration demonstrating the toxicity of this bacterium through ingestion.

Irregularly shaped epithelium, increased cytoplasmic vacuolization, nuclear chromatin condensation and cellular fragments with cytoplasmic and nuclear contents being released into the midgut lumen were typical characteristics of cell degeneration observed in midgut of *A. gemmatalis* fed on Bt toxin contaminated diet. Cellular degeneration in the midgut due to exposure to toxic compounds has been reported for *Alabama argillacea* Hübner (Lepidoptera: Noctuidae)^19^ and *Plutella xylostella* L. (Lepidoptera: Plutellidae)^36^. The release of cellular fragments, including nuclei, from epithelium into the midgut lumen observed in *A. gemmatalis* midgut after 2 h of Bt exposure can reduce the digestive capacity of insects as observed for *H. armigera*^37^ and suggests a detoxification response to the toxic effect of Bt and the cell death process^38^. The higher vacuolization in the *A. gemmatalis* digestive cells, exposed to the entomopathogen, suggests cell death^39^. The vacuole presence in the midgut cells is common in insects^40,41^, but its greater numbers in the cytoplasm has been characterized as autophagy^42,43^. The histological effects observed in *A. gemmatalis* midgut suggest an attempt to detoxify the entomopathogen infected cells.

Morphological changes observed in *A. gemmatalis* goblet cells showing deformed cells with numerous vacuoles in the cytoplasm^19^ are similar to those observed in other Lepidoptera^44^, suggesting a similar action mode of *B. thuringiensis* in these insects.

Bt induced the *A. gemmatalis* midgut peritrophic membrane rupture. This membrane was also destroyed in some midgut parts in *Alabama argillacea* (Lepidoptera: Noctuidae) fed on Bt cotton leaves^19^. Nutrient absorption is reduced due to the damage to the peritrophic membrane that plays a fundamental role in digestion^45^ and protects the epithelial cells from mechanical damage caused by the food bolus^45–47^, hindering pathogen entry and partitioning the digestion process^45,48^. The peritrophic membrane acts as a barrier against Bt toxins^49,50^ delaying contact with digestive cells^51^. However, these toxins can penetrate the peritrophic membrane^52^, bind to the receptors of the columnar cell microvilli and infect *A. gemmatalis* midgut epithelial cells.

Microvilli degeneration in *A. gemmatalis* columnar cells can be explained by the toxin effect on the cytoskeleton actin, therefore Bt can interact with membrane proteins during initial action stages^53^ inducing cytoplasm leakage into the midgut lumen^54^. Cellular protrusions released into the midgut lumen of *A. gemmatalis* caterpillars fed on Bt contaminated diet suggest a cytotoxic effect of this bacterium causing apoptosis, a morphological pattern of programmed cell death^55^. Elimination of cells by death^38^ would be a response to damage to midgut epithelial cells after Bt ingestion. Donut-shaped mitochondria were observed in the insect intestine exposed to Bt. This change in shape is caused by respiratory chain inhibition and is an early marker of cellular stress^56^ caused by entomopathogen.

The higher number of caspase-3 positive cells cleaved in the caterpillar midgut that ingested the bioinsecticide indicates apoptosis occurrence^43,57^. Cells showing a positive result for cleaved caspase-3 in the midgut of caterpillars fed on uncontaminated diet indicate normal cell renewal^43,58^.

The increase of proliferating cell numbers in the *A. gemmatalis* midgut after 8 hours of bacterial ingestion was indicated by anti-PH3 antibody, a mitosis cell-specific marker^59,60^. Damage to the insect’s digestive system by Bt toxins activating defensive responses were reported for *Heliothis virescens* Fabricius (Lepidoptera: Noctuidae)^61,62^. Epithelium regeneration with dead cells replaced by newly differentiated ones depends on the proliferation and differentiation of the regenerative cells and allows resistant insects to recover and survive after exposure to the biotic agent^63^. Cell replacement is important for the homeostatic maintenance of midgut integrity^64–66^. *Bombyx mori* Linnaeus (Lepidoptera: Bombycidae) responds to Bt infection with a regenerative mechanism^67,68^ by the asymmetric division of regenerative cells^44^. *Anticarsia gemmatalis* caterpillars do not have a continuous regenerative response as observed by the absence of cellular proliferation process in the midgut epithelium after 16 and 32 hours of Bt ingestion, possibly due to cell lysis and epithelial rupture providing a favorable medium for spore germination leading to severe septicemia and insect death^16,69^.

Toxins produced by *Bacillus thuringiensis* subsp*. kurstaki* strain HD-1 are harmful to *A. gemmatalis* at median lethal concentration and cause severe histological and ultrastructural changes degenerating the epithelium and causing the death of midgut epithelial cells in this insect.

## Material and Methods

### Insects

*Anticarsia gemmatalis* caterpillars were obtained from the insect biological control laboratory (LCBI) of the Universidade Federal de Viçosa, Viçosa, Minas Gerais, Brazil and maintained at 25 ± 2 °C, 75 ± 5% relative humidity and 12-hour photophase. These caterpillars were fed on an artificial diet consisting of 10 g of agar, 15.6 g of brewer’s yeast, 25 g of wheat germ, 25 g of soy protein, 31.2 g of beans, 12.5 g of casein, and 2.5 mL of vitamin solution (1.2% ascorbic acid, 0.03% calcium pantothenate, 0.015% niacin, 0.008%, riboflavin, 0.004% thiamine and 0.004% HCl)^70^. Twenty *A. gemmatalis* caterpillar groups were placed per polystyrene pot (15 × 9 cm) until pupa stage. Cleaning the pots and food replacement were performed every 48 hours. *Anticarsia gemmatalis* fourth instar larvae without amputations or apparent malformations were used in the bioassays.

### Toxicity test

*Bacillus thuringiensis* (Bt), subsp*. kurstaki* strain HD-1 Dipel® (Abbot Laboratories Chemical and Agricultural Products Division, North Chicago, IL, USA) was used in the toxicity test, diluted in 1 L of ultrapure water deionized in a Milli- Q (Millipore) to produce a stock solution, adjusting 100 g L^−1^ to obtain the required concentrations. The insecticidal efficacy was determined with lethal concentrations (LC_25_, LC_50_, LC_75_, LC_90_ and LC_99_) in the laboratory. Six Bt concentrations, besides the control (deionized ultrapure water) were adjusted in 10 mL stock solution (treatments and water): 0.1; 0.2; 0.4; 0.8; 1.6 and 3.2 mg mL^−1^ (w/v). Different concentrations of Bt were applied in 0.5 μL solution on 1 g of artificial diet. Fifty fourth instar *A. gemmatalis* caterpillars were used for each concentration individualized in Petri dishes (90 × 1.5 mm). The number of dead caterpillars after the exposure to Bt was counted every 12 h for 108 h.

### Histology

Twenty fourth-instar *A. gemmatalis* larvae were fed either on control or Bt contaminated diet with the median lethal concentration (LC_50_), for different time periods (2, 4, 8, 16 and 32 h) and cryoanesthesiated at −4 °C. The midgut was dissected in saline solution for insects (0,1 M NaCl + 0,1 M KH_2_PO_4_ + 0,1 M Na_2_HPO_4_) and transferred to Zamboni’s fixative solution^71^ for 12 h at 5 °C. The samples were dehydrated in increasing ethanol series (70, 80, 90 and 95%) and embedded in Leica historesin (Leica Biosystem Nussloch GmbH, Wetzlar, Germany) and sectioned at 3 μm thickness in Leica RM2255 microtome. Sections were stained with hematoxylin and eosin and analyzed under an Olympus BX-60 light microscope (Olympus Corporation, Tokyo, Japan).

### Ultrastructure

Twenty fourth-instar *A. gemmatalis* larvae were fed on Bt contaminated diet with the median lethal concentration (LC_50_) for 32 h and cryoanesthesiated at −4 °C. The midgut of these caterpillars was dissected and transferred to 2.5% glutaraldehyd in 0.2 M sodium cacodylate buffer, pH 7.2 containing 0.2 M sucrose for 4 h at room temperature. Samples were post-fixed in 1% osmium tetroxide in the same buffer for 2 h, washed in buffer, dehydrated in an increasing ethanol series (70, 80, 90 and 99%) and soaked in LR White resin (London Resin Company Ltd.). Ultra-fine sections (80–90 nm thick) were obtained with a diamond power razor in Power Tome-X ultramicrotome (Boeckeler Instruments, Tucson, AZ, USA), contrasted with 1% aqueous uranyl acetate and lead citrate^72^ and examined under transmission electron microscope Zeiss Libra 120 (Carl Zeiss, Jena, Germany).

### Immunofluorescence

Twenty *A. gemmatalis* caterpillar midguts, fed either on control or Bt contaminated diet with median lethal concentration (LC_50_) for 8, 16 and 32 h, were dissected in 0.1 M phosphate buffer sodium (PBS) (Sigma-Aldrich, St. Louis, MO, USA) and transferred to Zamboni fixing solution for 2 h. Then, the samples were washed with PBS containing 1% Triton X-100 (PBST) and incubated with cleaved anti-caspase 3 antibody (Cell Signaling Technology, Danvers, MA, USA) diluted at 1: 500 in PBS for detection of apoptosis, or with anti-histone H3 phosphoric (PH3) antibody (Cell Signaling Technology, Danvers, MA, USA) diluted at 1:400 in PBS for 24 h at 4 °C for cell proliferation detection. After incubation, the samples were washed in PBS and incubated with rabbit anti-IgG secondary antibody conjugated with fluorescein isotiosinate (Sigma-Aldrich, St. Louis, MO, USA) diluted 1: 500 in PBS for 24 h in the dark at 4 °C. The samples were then washed in PBS and the cell nuclei stained with TO-PRO-3 iodide (Life Technologies, Carlsbad, CA, USA) for 1 h. Samples were mounted on 50% sucrose glass slides and examined on Zeiss LSM510 META (Carl Zeiss, Jena, Germany) laser scanning confocal microscope.

### Statistical analysis

The lethal concentrations LC_25_, LC_50_, LC_90_, LC_99_ and confidence intervals were determined by regression based on probit-mortality concentration^73^ (Finney, 1971) with the PROC PROBIT procedure of the SAS User v. Program. 9.0 for Windows^74^.

## References

[1] Panizzi AR (2013). History and contemporary perspectives of the integrated pest management of soybean in Brazil. Neotrop. Entomol..

[2] Guedes RNC, Magalhaes LC, Cosme LV (2009). Stimulatory sublethal response of a generalist predator to permethrin: hormesis, hormoligosis, or homeostatic regulation?. J. Econ. Entomol..

[3] De Castro AA (2013). Survival and behavior of the insecticide-exposed predators *Podisus nigrispinus* and *Supputius cincticeps* (Heteroptera: Pentatomidae). Chemosphere.

[4] De Castro AA (2015). Demographic parameters of the insecticide-exposed predator *Podisus nigrispinus*: Implications for IPM. BioControl.

[5] Zanuncio JC, Batalha VC, Guedes RNC, Picanço MC (1998). Insecticide selectivity to *Supputius cincticeps* (Stal) (Het., Pentatomidae) and its prey *Spodoptera frugiperda* (J. E. Smith) (Lep., Noctuidae). J. Appl. Entomol..

[6] Tavares WS (2010). Selective effects of natural and synthetic insecticides on mortality of *Spodoptera frugiperda* (Lepidoptera: Noctuidae) and its predator *Eriopis connexa* (Coleoptera: Coccinellidae). J. Environ. Sci. Heal..

[7] Vryzas Z, Alexoudis C, Vassiliou G, Galanis K, Papadopoulou-Mourkidou E (2011). Determination and aquatic risk assessment of pesticide residues in riparian drainage canals in northeastern Greece. Ecotox. Environ. Safe..

[8] Damalas CA, Eleftherohorinos IG (2011). Pesticide exposure, safety issues, and risk assessment indicators. Int. J. Env. Res. Pub. He..

[9] Bishop AH, Johnnson C, Perani M (1999). The safety of *Bacillus thuringiensis* to mammalian investigated by oral and subcutaneous dosage. World J. Microb. Biot..

[10] Monnerat RG (2007). Screening of Brazilian *Bacillus thuringiensis* isolates active against *Spodoptera frugiperda*, *Plutella xylostella* and *Anticarsia gemmatalis*. Biol. Control.

[11] Almeida GD (2014). Cytotoxicity in the midgut and fat body of *Anticarsia gemmatalis* (Lepidoptera: Geometridae) larvae exerted by neem seeds extract. Isj-Invert. Surviv. J..

[12] Romeis J, Meissle M, Bigler F (2006). Transgenic crops expressing *Bacillus thuringiensis* toxins and biological control. Nat. Biotechnol..

[13] Van Rie J, Jansen S, Höfte H, Degheeled D, Van Mellaert H (1990). Receptors on the brush border membrane of the insect midgut as determinants of the specificity of *Bacillus thuringiensis* δ-endotoxins. Appl. Environ. Microb..

[14] Hofmann C (1998). Specificity of *Bacillus thuringiensis* δ-endotoxins is correlated with the presence of high affinity binding site in the brush border membrane of target insect midgut. P. Natl. A. Sci..

[15] Herrero S, González-Cabrera J, Tabashnik B, Ferré J (2001). Shared binding sites in Lepidoptera for *Bacillus thuringiensis* Cry1Ja and Cry1A toxins. Appl. Environ. Microb..

[16] Bravo A, Gill SS, Soberón M (2007). Mode of action of *Bacillus thuringiensis* Cry and Cyt toxins and their potential for insect control. Toxicon.

[17] Oestergaard J, Ehlers RU, Martínez-Ramírez AC, Real MD (2007). Binding of Cyt1Aa and Cry11Aa toxins of *Bacillus thuringiensis* serovar *israelensis* to brush border membrane vesicles of *Tipula paludosa* (Diptera: Nematocera) and subsequent pore formation. Appl. Environ. Microb..

[18] Grochulski P (1995). *Bacillus thuringiensis* CryIA(a) insecticidal toxin: crystal structure and channel formation. J. Mol. Biol..

[19] Sousa MEC (2010). Histopathology and ultrastructure of midgut of *Alabama argillacea* (Hübner) (Lepidoptera: Noctuidae) fed Bt-cotton. J. Insect Physiol..

[20] Lehane, M. J. & Billingsley, P. F. Biology of the insect midgut. Chapman & Hall, London (1996).

[21] Terra WR, Costa RH, Ferreira C (2006). Plasma membranes from insect midgut cells. An. Acad. Bras. Cienc..

[22] Turbeck B (1974). A study of the concentrically laminated concretions spherites in the regenerative cells of the midgut of Lepidopterous larvae. Tissue Cell.

[23] Serrão JE, Cruz-Landim C (1996). Ultrastructure of midgut endocrine cells in workers of stingless bee (Hymenoptera: Apidae: Meliponinae). Iheringia.

[24] Martins GF, Neves CA, Campos LAO, Serrão JE (2006). The regenerative cells during the metamorphosis in the midgut of bees. Micron.

[25] Andries JC, Beauvillain JC (1988). Ultrastructural study of cholecystokinin like immunoreactivity in endocrine cells of the insect midgut of *Nepa cinerea* (Insecta, Heteroptera): ultrastructure and genesis. Biol. Cell..

[26] Abdel-Razek AS (2002). Comparative histopathology of *Plodia interpunctella* (Lepidoptera: Pyralidae) and *Tribolium castaneum* (Coleoptera: Tenebrionidae) as affected by *Bacillus thuringiensis* varieties Indiana or Morrison. Archives of Phytopathology and Plant Protection.

[27] Sutherland PW, Harris MO, Markwick NP (2003). Effects of starvation and the *Bacillus thuringiensis* endotoxin Cry1Ac on the midgut cells, feeding behavior, and growth of light brown apple moth larvae. Ann. Entomol. Soc. Am..

[28] Hong-Wei Y, Cai-Ying J, Gong-Yin Y, Cui H, Yu-Fa P (2008). Toxicological assessment of pollen from different Bt rice lines on *Bombyx mori* (Lepidoptera: Bombyxidae). Environ. Entomol..

[29] Federici BA (2003). Insecticidal bacteria: an overwhelming success for invertebrate pathology. J. Invertebr. Pathol..

[30] Sanahuja G, Banakar R, Twyman RM, Capel T, Chrstou P (2011). *Bacillus thuringiensis*: a century of research, development and commercial applications. Plant Biotechnol. J..

[31] Wermelinger ED, Zanuncio JC, Rangel EF, Cecon PR, Rabinovitch L (2000). Toxicity of *Bacillus* species to larvae of *Lutzomyia longipalpis* (L. & N.) (Diptera: Psychodidae: Phlebotominae). Anais da Sociedade Entomológica do Brasil.

[32] Elleuch J (2016). Toxin stability improvement and toxicity increase against dipteran and lepidopteran larvae of *Bacillus thuringiensis* crystal protein Cry2Aa. Pest Manag. Sci..

[33] Da Silva KF, Spencer TA, Crespo ALB, Siegfried BD (2016). Susceptibility of *Spodoptera frugiperda* (Lepidoptera: Noctuidae) field populations to the Cry1F *Bacillus thuringiensis* insecticidal protein. Fla. Entomol..

[34] Regode V, Kuruba S, Mohammad AS, Sharma HC (2016). Isolation and characterization of gut bacterial proteases involved in inducing pathogenicity of *Bacillus thuringiensis* toxin in cotton bollworm, *Helicoverpa armigera*. Front. Microbiol..

[35] Vineela V, Nataraj T, Reddy G, Devi PSV (2017). Enhanced bioefficacy of *Bacillus thuringiensis* var. *kurstaki* against *Spodoptera litura* (Lepidoptera: Noctuidae) through particle size reduction and formulation as a suspension concentrate. Biocontrol Sci. Techn..

[36] Ribeiro LMS (2013). Midgut histopathology of resistant and susceptible *Plutella xylostella* exposed to commercial formulations of *Bacillus thuringiensis*. B. Insectol..

[37] Barbeta BL, Marshal AT, Gillon A, Craik DJ, Marlyn AA (2008). Plant cyclotides disrupt epithelial cell in the midgut of Lepidoptera larvae. P. Natl. Acad. Sci. Usa..

[38] Santos MC, Junqueira AMR, de Sá VGM, Zanuncio JC, Serrão JE (2015). Effect of silicon on the morphology of the midgut and mandible of tomato leafminer *Tuta absoluta* (Lepidoptera: Gelechiidae) larvae. Isj-Invert. Surviv. J..

[39] Hariri M (2000). Biogenesis of multilamellar bodies via autophagy. Mol. Biol. Cell..

[40] Alves SN, Serrão JE, Melo AL (2010). Alterations in the fat body and midgut of *Culex quinquefasciatus* larvae following exposure to different insecticides. Micron.

[41] Fernandes KM (2015). Imidacloprid impairs the post-embryonic development of the midgut in the yellow fever mosquito *Stegomyia aegypti* (=*Aedes aegypti*). Med. Vet. Entomol..

[42] Levine B, Klionsky DJ (2004). Development by self-digestion: molecular mechanisms and biological functions of autophagy. Dev. Cell.

[43] Santos DE, Azevedo DO, Campos LAO, Zanuncio JC, Serrão JE (2015). *Melipona quadrifasciata* (Hymenoptera: Apidae) fat body persists through metamorphosis with a few apoptotic cells and an increased autophagy. Protoplasma.

[44] Loeb MJ, Martin PAW, Hakim RS, Goto S, Takeda M (2001). Regeneration of cultured midgut cells after exposure to sublethal doses of toxin from two strains of *Bacillus thuringiensis*. J. Insect Physiol..

[45] Terra WR (1988). Physiology and biochemistry of insect digestion, an evolutionary perspective. Braz. J. Med. Biol. Res..

[46] De Priester W (1971). Ultrastructure of the midgut epithelial cells in the fly *Calliphora erythrocephala*. Journal of Ultrastructure Research.

[47] Ryerse JS, Purcell JP, Sammons RD, Lavrik PB (1992). Peritrophic membrane structure and formation in the larva of a moth, *Heliothis*. Tissue Cell.

[48] Terra WR (2001). The origin and functions of the insect peritrophic membrane and peritrophic gel. Arch. Insect Biochem..

[49] Hayakawa T, Shitomi Y, Miyamoto K, Hori H (2004). GalNAc pretreatment inhibits trapping of *Bacillus thuringiensis* Cry1Ac on the peritrophic membrane of *Bombyx mori*. Febs Lett..

[50] Rodrigo-Simon A (2006). Lack of detrimental effects of *Bacillus thuringiensis* Cry toxins on the insect predator *Chrysoperla carnea*: a toxicological, histopathological, and biochemical analysis. Appl. Environ. Microb..

[51] Wu K (2016). Gut immunity in Lepidopteran insects. Developmental & Comparative Immunology.

[52] Adang MJ, Spence KD (1981). Surface morphology of peritrophic membrane formation in the cabbage looper, *Trichoplusia ni*. Cell Tissue Res..

[53] Griffitts JS (2003). Resistance to a bacterial toxin is mediated by removal of a conserved glycosylation pathway required for toxine host interactions. J. Biol. Chem..

[54] Qi Z, Shi B, Hua Z, Zhang Y, Wua W (2011). Ultrastructural effects of Celangulin V on midgut cells of the oriental armyworm, *Mythimna separata* walker (Lepidoptera: Noctuidae). Ecotox. Environ. Safe..

[55] Ihara T, Tsukiko YMS, Ueno HOY (1998). The process of ultrastructural changes from nuclei to apoptotic body. Virchows Arch..

[56] Ahmad T (2013). Computational classification of mitochondrial shapes reflects stress and redox state. Cell Death Dis..

[57] Vishwanathreddy H, Bhat GG, Inamdar SR, Gudihal RK, Swamy BM (2014). *Sclerotium rolfsii* lectin exerts insecticidal activity on *Spodoptera litura* larvae by binding to membrane proteins of midgut epithelial cells and triggering caspase-3-dependent apoptosis. Toxicon.

[58] Franzetti E (2012). Autophagy precedes apoptosis during the remodeling of silkworm larval midgut. Apoptosis.

[59] Su TT, Sprenger F, DiGregorio PJ, Campbell SD, O’Farrell PH (1998). Exit from mitosis in *Drosophila* syncytial embryos requires proteolysis and cyclin degradation, and is associated with localized dephosphorylation. Gene Dev..

[60] Idikio HA (2006). Spindle checkpoint protein hMad2 and histone H3 phosphoserine 10 mitosis marker in pediatric solid tumors. Anticancer Res..

[61] Forcada C, Alcacer E, Garcera MD, Tato A, Martinez R (1999). Resistance to *Bacillus thuringiensis* Cry1Ac toxin in three strains of *Heliothis virescens*: proteolytic and SEM study of the larval midgut. Arch. Insect Biochem..

[62] Martinez-Ramirez AC, Gould F, Ferre J (1999). Histopathological effects and growth reduction in a susceptible and a resistant strain of *Heliothis virescens* (Lepidoptera: Noctuidae) caused by sublethal doses of pure Cry1A crystal proteins from *Bacillus thuringiensis*. Biocontrol Sci. Techn..

[63] Castagnola A, Jurat-Fuentes JL (2016). Intestinal regeneration as an insect resistance mechanism to entomopathogenic bacteria. Curr. Opin. Insect Sci..

[64] Okuda K (2007). Cell death and regeneration in the midgut of the mosquito, *Culex quinquefasciatus*. J. Insect Physiol..

[65] Rost-Roszkowska MM, Machida R, Fukui M (2010). The role of cell death in the midgut epithelium in *Filientomon takanawanum* (Protura). Tissue Cell.

[66] Rost-Roszkowska MM, Poprawa I, Chachulska-Zymeka A (2010). Apoptosis and autophagy in the midgut epithelium of *Acheta domesticus* (Insecta, Orthoptera, Gryllidae). Zool Sci..

[67] Chiang AS, Yen DF, Peng WK (1986). Defense reaction of midgut epithelial cells in the rice moth larva (*Corcyra cephalonica*) infected with *Bacillus thuringiensis*. J. Invertebr. Pathol..

[68] Spies AG, Spence KD (1985). Effect of sublethal *Bacillus thuringiensis* crystal endotoxin treatment on the larval midgut of a moth, *Manduca sexta*. Tissue Cell.

[69] De Maagd RA, Bravo A, Crickmore N (2001). How *Bacillus thuringiensis* has evolved specific toxins to colonize the insect world. Trends in Genetics.

[70] Greene GL, Leppla NC, Dickerson WA (1976). Velvetbean caterpillar: a rearing procedure and artificial medium. J. Econ. Entomol..

[71] Stefanini M, Martino CD, Zamboni L (1967). Fixation of ejaculated spermatozoa for electron microscopy. Nature.

[72] Reynolds ES (1963). The use of lead citrate at high pH as an electron-opaque stain in electron microscopy. J. Cell Biol..

[73] Finney, D. J. Probit Analysis. Cambridge University Press (1971).

[74] SAS Institute. The SAS System for Windows, release 9.0. SAS Institute, Cary, N.C (2002).

